# Indications and prescriptions of penicillins in a population of Colombia: A cross-sectional study

**DOI:** 10.1016/j.bjid.2025.104572

**Published:** 2025-07-16

**Authors:** Luis Fernando Valladales-Restrepo, Brayan Stiven Aristizábal-Carmona, Luisa María Londoño-Toro, Mariavictoria del Valle Jaramillo-Lima, Mariana Osorno-Ríos, Jorge Enrique Machado-Alba

**Affiliations:** a Universidad Tecnológica de Pereira-Audifarma S.A, Grupo de Investigación en Farmacoepidemiología y Farmacovigilancia, Pereira, Risaralda, Colombia; b Fundación Universitaria Autónoma de las Américas, Facultad de Medicina, Grupo de Investigación Biomedicina, Pereira, Colombia; c Fundación Universitaria Autónoma de las Américas, Facultad de Medicina, Semillero de Investigación en Farmacología Geriátrica, Grupo de Investigación Biomedicina, Pereira, Risaralda, Colombia

**Keywords:** Amoxicillin, Colombia, Common cold, *Helicobacter pylori*, Inappropriate prescribing, Penicillins

## Abstract

Inappropriate use of antibiotics plays a key role in increasing bacterial resistance. The aim was to determine the prescription patterns and approved and unapproved indications for the use of penicillins in a group of patients from Colombia. This was a cross-sectional study on the use of penicillins in outpatients. The subjects were identified from a population-based drug dispensing database. Approved and unapproved indications were determined from records of the Food and Drug Administration (FDA) of the United States and the National Institute of Food and Drug Surveillance (INVIMA) of Colombia. Descriptive and multivariate analyses were performed. A total of 137,070 patients were identified; the average age was 35.8 ± 23.5 years, and 56.2 % were women. Amoxicillin (73.4 %), dicloxacillin (11.7 %) and sultamicillin (6.0 %) were the most prescribed penicillins, mainly for upper respiratory tract infections (43.0 %). In 68.9 % cases, penicillins were used for approved indications, especially to treat *Helicobacter pylori* (17.3 %). In 31.1 % of cases, penicillin prescriptions were used for unapproved indications (acute rhinopharyngitis: 8.1 %). Patients with skin and soft tissue infections (aOR = 2.82; 95 % CI 2.57‒3.09), with lower respiratory tract infections (aOR = 2.02; 95 % CI 1.89‒2.16), and those treated with dicloxacillin (aOR = 2.84; 95 % CI 2.07‒3.89) were more likely to be prescribed penicillins for unapproved indications. Amoxicillin was the most widely used penicillin in outpatients. Penicillins were frequently used for unapproved indications not recommended by drug regulatory agencies.

## Introduction

The discovery of penicillin and its therapeutic potential in the management of bacterial infections is one of the greatest advances in therapeutic medicine.^1^^,^^2^ Penicillin ushered in the age of antibiotics^1^ with its extensive use beginning in the 1940s, and it is still valuable today.^1^^,^^2^ Penicillins are bactericidal and effective over a spectrum of gram-positive, gram-negative, and anaerobic microorganisms.^2^ The appearance of resistant bacterial strains has limited their use in recent years;^3^^,^^4^ however, penicillins are still very useful in the management of some respiratory tract infections (e.g., tonsillitis, pneumonia), skin and soft tissue infections (e.g., cellulitis, erysipelas), and gastrointestinal tract infections (*Helicobacter pylori*, diarrhea of bacterial etiology), among others.^5^^,^^6^ Penicillins are currently the most widely used antibiotics in Colombia^7^ and worldwide.^3^^,^^8^

Antimicrobial resistance is among the top 10 threats to global health and has significant socioeconomic and public health impacts.^9^ These impacts are usually more serious in low- and middle-income countries.^3^^,^^4^ Various factors are involved in their effects on public health, but the excessive or improper use of antibiotics is the main factor leading to the emergence of antimicrobial resistance.^9^ The inappropriate use of antibiotics is commonly due to incorrect drug selection or determination of a dose and duration of treatment that do not correspond to the recommendations of clinical practice guidelines.^4^^,^^10^ Sociodemographic conditions and factors of the prescriber and/or patients contribute to the inappropriate use of antibiotics.^11^ For this reason, the World Health Organization (WHO) constantly promotes the rational use of antibiotics in populations.^10^ Multidrug-resistant microorganisms are responsible for 700,000 deaths worldwide each year and are projected to cause 10 million deaths by 2050.^12^ These infections are associated with increased costs of care, prolonged hospital stays, and increased mortality.^12^^,^^13^

The Colombian health system has a health benefits plan that provides universal coverage to all people through two paths (contributory and subsidized). The contributory regimen is paid for by people with a work contract and their employers, as well as independent workers with the ability to pay and people who are retired. The subsidized regimen is paid for by the state. The health benefit plan is the same for both regimens and includes several penicillins at different dosages.^14^ The access route to the Colombian health system is through the general practitioner. They can refer patients to being evaluated by specialist doctors. Studies have been conducted in the country on patients with skin and soft tissue infections, as well as on patients with *Helicobacter pylori* infections, and evidence of inappropriate use of antibiotics has been found.^15^^,^^16^ However, the specific use of penicillins in the outpatient setting, the degree of adherence to the recommendations of drug regulatory agencies, and their use in approved and unapproved indications in Colombia are unknown. The objective of this study was to determine the prescription patterns and approved and unapproved indications for the use of penicillins in a group of patients from Colombia.

## Materials and methods

### Study design and patients

An observational cross-sectional study was conducted to establish prescription patterns and approved and unapproved indications for the use of penicillins in outpatients. The subjects were identified from a population-based drug dispensing database that includes information from approximately 9.5 million people affiliated with the Colombian health system through four health insurance companies, corresponding to approximately 25.3 % of the active affiliated population on the contributory or payment regimen and 13.1 % of the population on the state-subsidized regimen, which together comprise 18.8 % of the Colombian population. The drug dispensing database contains sociodemographic variables (sex, age, city of dispensing, and affiliation regime), pharmacological (medication, pharmaceutical form, number of drugs, dose, and prescribing physician), and primary and secondary diagnoses (codes and description of the International Classification of Diseases version-10 [ICD-10]).^17^^,^^18^ More than 200 pharmacoepidemiology research studies have been published using this database.^17^^,^^18^

**Fig. 1:**
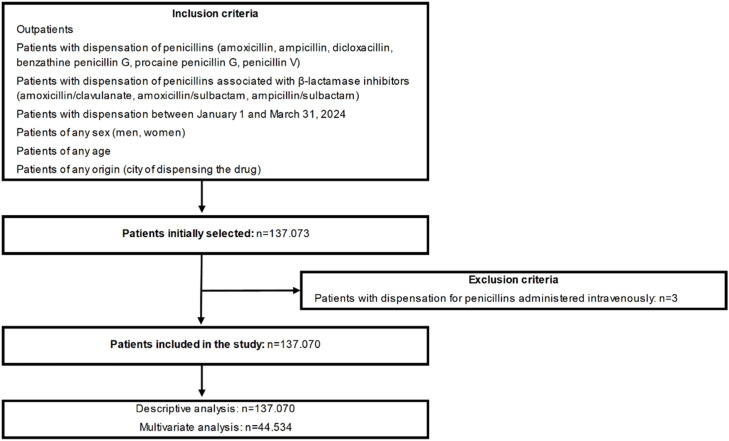
The flow diagram of the study.

Patients with a first dispensation of penicillins (amoxicillin, ampicillin, dicloxacillin, benzathine penicillin G, procaine penicillin G and penicillin V) or penicillins associated with β-lactamase inhibitors (amoxicillin/clavulanate, amoxicillin/sulbactam, ampicillin/sulbactam [sultamicillin]) between January 1 and March 31, 2024, were included. Patients of any sex (men, women), age and origin (city of dispensing the drug) were selected and treated via outpatient medical consultation. Patients who received pharmaceuticals through intravenous administration or at the hospital were excluded. The flow diagram of the study is shown in Fig. 1.

### Variables

Using data on the drug consumption of the affiliated population systematically obtained by the dispensing company Audifarma SA,^17^ a database was designed that included the following variables:

a) Sociodemographic: Age, sex, affiliation regime with the health system (contributory or subsidized) and place of origin (city of dispensing the drug). The place of origin was classified by departments according to the regions of Colombia according to the classification of the National Administrative Department of Statistics (DANE) of Colombia as follows: Bogotá-Cundinamarca region, Caribbean region, Central region, Pacific region and Eastern region- Amazon-Orinoquía (Supplementary Table 1). Origin was also classified into capital cities and municipalities.
b) Comorbidities: Comorbidities were identified from the main diagnoses reported using the codes of the International Classification of Diseases version-10 (ICD-10). These disorders were categorized into cardiovascular, rheumatological, neurological/psychiatric, endocrine and oncological disorders, among others.
c) Pharmacological:
  Type of prescriber: General practitioner, medical specialties (internal medicine, pediatrics), surgical specialties (general surgery, orthopedics) and dentistry.
  Penicillin type: Natural (benzathine penicillin G, procaine penicillin G, penicillin V), aminopenicillins (amoxicillin, ampicillin), dicloxacillin, and penicillins associated with β-lactamase (amoxicillin/clavulanate, amoxicillin/sulbactam and sultamicillin [oral combination of ampicillin/sulbactam]); pharmaceutical form (tablet or capsule, powder to be reconstituted as an oral solution or powder to be reconstituted for injection); and dose. The Defined Daily Dose (DDD) was the unit of measurement for the use of drugs, according to the recommendations of the WHO, and was expressed as DHD (defined daily dose per 1000 inhabitants per day).^19^
  Indications: The main diagnosis associated with each prescription of penicillins was determined according to the ICD-10 codes, and whether the indication was approved or not approved was determined according to the records of the United States Food and Drug Administration (FDA) and the National Surveillance Institute. of Medicines and Foods (INVIMA) of Colombia. The approved indications are shown in Supplementary Table 2.^5^^,^^6^
d) Comedications:
  Concomitant antibiotics with penicillins and antibiotics received in the 30-days prior to the index date were grouped into the following categories: a) Macrolides (azithromycin, clarithromycin, erythromycin), b) Tetracyclines (doxycycline, minocycline, tetracycline), c) Cephalosporins (cephalexin, cephradine, cefuroxime, cefpodoxime), d) Aminoglycosides (amikacin, gentamicin), e) Nitroimidazoles (metronidazole, secnidazole, tinidazole), and f) Urinary antiseptics (nitrofurantoin, fosfomycin).

Others: a) Antidiabetics, b) Antihypertensives and diuretics, c) Lipid-lowering drugs, d) Antiulcer drugs, e) Antidepressants, f) Anxiolytics and hypnotics (benzodiazepines and Z drugs), g) Thyroid hormones, h) Antipsychotics, i) Antiepileptics, j) Antihistamines, and k) Analgesics and anti-inflammatories.

### Ethics statement

The protocol was endorsed by the Research Ethics Committee of the University of Colombia in the category of “research without risk” (approval code: 14-130223). The principles of confidentiality of information established by the Declaration of Helsinki were respected.

### Statistical analysis

The data were analyzed with the statistical package SPSS Statistics, version 26.0 for Windows (IBM, USA).^20^ Descriptive analysis was performed with frequencies and proportions for the qualitative variables and means and standard deviations for the quantitative variables. An exploratory multivariate analysis was performed using binary logistic regression. The dependent variable was the use of penicillins for unapproved indications (Yes/No). The independent variables (covariates) were those that showed statistical significance with respect to the dependent variable in the bivariate analyses. This analysis was performed using Pearson's χ^2^ test. The intro method was used to select the variables in the multivariate model. Crude Odds Ratios (cOR) and adjusted Odds Ratios (aOR) with 95 % Confidence Intervals are presented; *p* < 0.05 was considered statistically significant. The model was adjusted for sex, age, origin and type of prescribing physician.

## Results

### Sociodemographic data

A total of 137,070 patients who received the first prescription of a penicillin distributed across 227 different cities were identified. A total of 56.2 % (*n* = 77,087) of the participants were women, and the average age was 35.8-years (range: 0.0‒104.0 years). A total of 26.0 % (*n* = 35,637) were under 18-years old, 26.4 % (*n* = 36,165) were between 18 and 39-years-old, 29.5 % (*n* = 40,379) were between 40 and 64-years-old, and 11.8 % (*n* = 16,202) were 65-years or older. The patients were predominantly from the Caribbean region and in capital cities and were affiliated mainly with the contributory scheme within the country's health system (Table 1).

**Table 1:** Sociodemographic variables, comorbidities and co-medications, of a group of patients treated with penicillins, Colombia.

Variables	Total	
	*n* = 137,070	%
**Sociodemographic**	‒	‒
Women	77,087	56.2
Age, mean ± Standard deviation	35.8 ± 23.5	
Origin (geographical regions)	‒	‒
Caribbean region	60,094	43.8
Bogotá-Cundinamarca region	37,950	27.7
Central region	18,742	13.7
Pacific region	13,952	10.2
Eastern-Orinoquia-Amazonia region	6332	4.6
Origin (capital cities)	76,512	55.8
Origin (municipalities)	60,558	44.2
Health system affiliation regime	‒	‒
Contributory	78,524	57.3
Subsidized	58,546	42.7
**Comorbidities**	‒	‒
Cardiovascular	19,527	14.2
Endocrine	11,422	8.3
Digestive	6771	4.9
Neurological or psychiatric	3455	2.5
Respiratory	2296	1.7
Rheumatological	2051	1.5
Neoplasms	1172	0.9
**Comedications**	‒	‒
Analgesics and anti-inflammatories	97,324	71.0
Antihistamines	33,613	24.5
Antiulcer drugs	20,865	15.2
Antihypertensives and diuretics	16,256	11.9
Bronchodilators and inhaled corticosteroids	15,584	11.4
Lipid-lowering drugs	13,100	9.6
Systemic corticosteroids	11,036	8.1
Antidiabetics	6767	4.9
Antispasmodics	5805	4.2
Thyroid hormone	5298	3.9
**Systemic antibiotics concomitant**	‒	‒
Macrolides	6633	4.8
Nitroimidazoles	5301	3.9
Fluoroquinolones	1721	1.3
Tetracyclines	1523	1.1
Aminoglycosides	1120	0.8
Cephalosporins	942	0.7
Lincosamides	538	0.4
Urinary antiseptics	281	0.2
Sulfonylureas	241	0.2
Bismuth salts	167	0.1
Rifaximin	88	0.1

### Comorbidities

A total of 26.1 % (*n* = 35,745) of the patients had chronic pathology, predominantly cardiovascular pathology (Table 1). The 10 most common comorbidities were arterial hypertension (*n* = 19,106; 13.9 %), hypothyroidism (*n* = 5298; 3.9 %), chronic gastritis (*n* = 4883; 3.6 %), diabetes mellitus (*n* = 4572, 3.3 %), dyslipidemia (*n* = 1650, 1.2 %), asthma (*n* = 1437, 1.0 %), benign prostatic hyperplasia (*n* = 1383, 1.0 %), irritable bowel syndrome (*n* = 1310, 1.0 %), cancer (*n* = 1087, 0.8 %) and anxiety disorders (*n* = 1037, 0.8 %).

### Pharmacological

Penicillins were prescribed mainly by general medicine practitioners (*n* = 127,058; 92.7 %), followed by dentists (*n* = 4572; 3.3 %), clinical specialists (*n* = 2947; 2.1 %), surgeons (*n* = 2277; 1.7 %) and nurses (*n* = 216; 0.2 %). The most prescribed antibiotic was amoxicillin, followed by dicloxacillin and sultamicillin (Table 2). Tablets or capsules were the most used pharmaceutical forms (*n* = 101,745; 74.2 %), followed by powder to reconstitute into oral solution (*n* = 29,908; 21.8 %) and powder to reconstitute into injectable solution (*n* = 5797; 4.2 %). Table 2 shows the pattern of penicillin use, frequency of use, prescribed dose, and distribution by sex and age. A total of 4.3 % (*n* = 5854) of the patients received antibiotics, mainly cephalosporins (*n* = 2039; 1.5 %) and macrolides (*n* = 948; 0.7 %), within the 30-days prior to the index date. A total of 11.6 % (*n* = 15,909) of the patients received another antibiotic concomitantly with penicillin, mainly macrolides (Table 1). The most frequent comedications were analgesics and anti-inflammatories (Table 1).

**Table 2:** Pattern of use of penicillins, frequency of use, distribution by sex and age, and prescribed dose, in a group of patients from Colombia.

Antibiotic	*n* = 137,070	%	Prescribed dose (mg/day)			Sex		Age
			Mean (SD)	Mode	DHD	F ( %)	M ( %)	Mean (SD)
Amoxicillin	100,573	73.4	2080.2 ± 377.1	2000	30.1	56.5	43.5	34.1 ± 23.5
Dicloxacillin	15,984	11.7	1509.1 ± 138.9	1500	3.4	54.2	45.8	40.6 ± 22.2
Sultamicillin	8203	6.0	1411.1 ± 242.5	1500	1.9	59.9	40.1	47.6 ± 23.2
Penicillin G benzathine	5689	4.2	1986,359.6 ± 570,374.7	2400,000	‒	47.8	52.2	36.6 ± 18.5
Ampicillin	3798	2.8	2027.7 ± 343.2	2000	0.8	67.1	32.9	37.9 ± 23.0
Amoxicillin/Clavulanate	2839	2.1	1780.4 ± 422.9	1750	0.8	52.3	47.7	31.6 ± 26.8
Amoxicillin/Sulbactam	337	0.2	2285.0 ± 427.1	2625	0.1	57.9	42.1	49.3 ± 21.5
Penicillin G procaine	128	0.1	815,625.0 ± 161,896.9	800,000	‒	51.6	48.4	32.4 ± 22.4
Penicillin V	29	0.0	1941.2 ± 166.1	2000	0.0	41.4	58.6	34.2 ± 24.6

M, Male; F, Female; SD, Standard Deviation; DHD, Defined Daily Dose per 1000 inhabitants per day.

A total of 32.5 % (*n* = 44,534) of the patients had diagnoses related to infections, mainly of the upper respiratory tract (19,141/44,534; 43.0 %), gastrointestinal tract (*n* = 8317; 18.7 %), skin and soft tissues (*n* = 5045; 11.3 %), lower respiratory tract (*n* = 4310; 9.7 %) and dental tissue (*n* = 3447; 7.7 %). Among the patients who were diagnosed, 68.9 % (*n* = 30,671/44,534) used antibiotics for approved indications, mainly in the management of *Helicobacter pylori* infections (Table 3). The main eradication regimens were the combination of a Proton Pump Inhibitor (PPI) + amoxicillin + clarithromycin (*n* = 2770/7699; 36.0 %), followed by PPI + amoxicillin + clarithromycin + metronidazole (*n* = 1485; 19.3 %), PPI + amoxicillin + metronidazole (*n* = 1006; 13.1 %), PPI + amoxicillin + levofloxacin (*n* = 932; 12.1 %) and PPI + amoxicillin + doxycycline (*n* = 494; 6.4 %). A total of 31.1 % (*n* = 13,863) of the patients received penicillins for unapproved indications, particularly acute rhinopharyngitis (Table 3). The use of penicillins for unapproved indications was more common in municipalities than in capital cities (37.8 % vs. 26.8 %; *p* < 0.001). Table 3 shows the main approved and nonapproved indications for the prescription of penicillins.

**Table 3:** Use of penicillins in approved and unapproved indications, in a group of patients, Colombia.

Variables	Total		Capital cities		Municipalities	
	*n* = 44,534	%	*n* = 26,874	%	*n* = 17,660	%
**Approved**	30,671	68.9	19,680	73.2	10,991	62.2
*Helicobacter pylori* infection	7699	17.3	5420	20.2	2279	12.9
Acute tonsillitis	5469	12.3	3388	12.6	2081	11.8
Acute otitis media	4637	10.4	3167	11.8	1470	8.3
Acute sinusitis	1979	4.4	1390	5.2	589	3.3
Unspecified bacterial lower respiratory infections	1472	3.3	652	2.4	820	4.6
Periodontitis	1470	3.3	1073	4.0	397	2.2
Periapical abscess	1441	3.2	764	2.8	677	3.8
Cellulitis or erysipelas	1332	3.0	802	3.0	530	3.0
Unspecified bacterial upper respiratory infections	1272	2.9	592	2.2	680	3.9
Strep pharyngitis	1164	2.6	844	3.1	320	1.8
Pneumonia	846	1.9	466	1.7	380	2.2
Syphilis	552	1.2	335	1.2	217	1.2
Gingivitis	203	0.5	142	0.5	61	0.3
Cellulitis and/or abscess in mouth	141	0.3	85	0.3	56	0.3
Exacerbated chronic obstructive pulmonary disease	121	0.3	58	0.2	63	0.4
Others (*n* = 35)	873	2.0	502	1.9	371	2.1
**Not approved**	13,863	31.1	7194	26.8	6669	37.8
Common cold	3589	8.1	1761	6.6	1828	10.4
Acute bronchitis	1502	3.4	951	3.5	551	3.1
Skin abscess	1325	3.0	680	2.5	645	3.7
Urinary tract infection	1238	2.8	611	2.3	627	3.6
Unspecified fever	968	2.2	339	1.3	629	3.6
Otitis externa	730	1.6	416	1.5	314	1.8
Skin wounds	410	0.9	160	0.6	250	1.4
Gastroenteritis	361	0.8	204	0.8	157	0.9
Ingrown toenail	298	0.7	203	0.8	95	0.5
Burn	272	0.6	134	0.5	138	0.8
Impetigo	219	0.5	90	0.3	129	0.7
Cellulitis or erysipelas	215	0.5	116	0.4	99	0.6
Vaginitis ‒ Vulvitis ‒ Vulvovaginitis	215	0.5	121	0.5	94	0.5
Unspecified viral infections	209	0.5	76	0.3	133	0.8
Bronchiolitis	203	0.5	115	0.4	88	0.5
Others (*n* = 88)	2109	4.7	1217	4.5	892	5.1

### Multivariate analysis

Exploratory logistic regression revealed that unapproved indications were more common in patients under 18-years of age (Aor = 1.87; 95 % CI 1.78‒1.96), those from municipalities (aOR = 1.51; 95 % CI 1.44‒1.57), those with recent use of antibiotics (aOR = 2.49; 95 % CI 2.26‒2.74), those diagnosed with lower respiratory tract infections (aOR = 2.02; 95 % CI 1.89‒2.16) or skin and soft tissue infections (aOR = 2.82; 95 % CI 2.57‒3.09), those managed by general medicine practitioners (aOR = 1.37; 95 % CI 1.25‒1.51) and those treated with dicloxacillin (aOR = 2.84; 95 % CI 2.07‒3.89) (Table 4).

**Table 4:** Binary logistic regression of variables related to the use of penicillins in unapproved indications, Colombia.

Variables	cOR	95 % CI		p	aOR	95 % CI		p
		Lower	Upper			Lower	Upper	
Woman (Yes/No)	0.870	0.836	0.906	<0.001	0.975	0.933	1.019	0.254
Age < 18-years (Yes/No)	1.589	1.522	1.660	<0.001	1.868	1.781	1.960	<0.001
Origin of municipalities (Yes/No)	1.660	1.594	1.729	<0.001	1.505	1.440	1.572	<0.001
Prescription by general medicine (Yes/No)	1.688	1.547	1.842	<0.001	1.374	1.253	1.506	<0.001
Use of antibiotics in the last 30-days (Yes/No)	2.360	2.159	2.579	<0.001	2.491	2.263	2.742	<0.001
Lower respiratory tract infection (Yes/No)	1.725	1.618	1.839	<0.001	2.018	1.885	2.160	<0.001
Skin and soft tissue infections (Yes/No)	5.854	5.496	6.235	<0.001	2.816	2.567	3.089	<0.001
Aminopenicillins (Yes/No)	0.324	0.309	0.339	<0.001	0.786	0.579	1.067	0.123
Aminopenicillins + β-lactamase inhibitor (Yes/No)	1.649	1.533	1.774	<0.001	1.288	0.943	1.760	0.111
Dicloxacillin (Yes/No)	6.246	5.842	6.677	<0.001	2.840	2.073	3.892	<0.001
Natural penicillins (Yes/No)	0.863	0.790	0.943	0.001	1.015	0.754	1.368	0.920

cOR, Crude Odds Ratios; aOR, Adjusted Odds Ratio; CI, Confidence Interval.

## Discussion

This study revealed the prescription patterns of penicillins and their use for approved and unapproved indications in outpatients from different geographic regions of Colombia. A predominance of amoxicillin prescriptions was observed, and some therapeutic behaviors were not consistent with the recommendations of the clinical practice guidelines. Studies with real-world evidence provide insight into how drugs are used in populations, allowing the use of interventions to improve the quality of prescriptions if their use is not appropriate. The WHO has estimated that more than half of the world's medicines are improperly prescribed, dispensed or sold.^10^ However, the implementation of antimicrobial optimization programs in Colombia aims to improve the rational use of antibiotics and reduce antimicrobial resistance.^21^^,^^22^

The consumption of penicillins in this study was greater than that reported in the WHO and European registries.^3^^,^^8^ According to the WHO Global Antimicrobial Resistance and Use Surveillance System (GLASS), which includes data from 27 countries on different continents, the consumption of penicillins is 7.1 DDD per 1000 inhabitants per day.^3^ According to the European Surveillance of Antimicrobial Consumption Network (ESAC—Net), in outpatients from 28 European countries, the consumption of penicillins is 8.0 DDD per 1000 inhabitants/day.^8^ Amoxicillin was the most commonly used penicillin in this study, which is consistent with findings in Colombia^7^ and worldwide.^3^^,^^8^ The high prescription of amoxicillin may be due to its low cost since all pharmaceutical forms of amoxicillin are covered by the Colombian Health System.^14^ Additionally, amoxicillin is an essential antibiotic and belongs to the WHO “Access” group of antibiotics, which are recommended as first- or second-line empirical treatment options for many common infections.^23^ In contrast, the high prescription rate could also indicate an overuse of antibiotics or their use for unapproved indications.^10^^,^^24^ Furthermore, it is important to highlight that resistance to this penicillin is common, which could lead to therapeutic failure in some patients.^25^

The prescription of penicillins for unapproved indications was found in 31.1 % of patients, which is greater than the rate reported in other international studies with real-world evidence.^26^, ^27^, ^28^ In China, 6.6 % of penicillin prescriptions were documented as inappropriate,^26^ whereas in Canada and the U.S., this figure is 18.3 % and 19.5 %, respectively.^27^^,^^28^ However, in a meta-analysis on the prescription of antibiotics in primary care in low- and middle-income countries, most patients received antibiotics inappropriately (79.7 %; range: 7.9 %‒100 %).^4^ In Colombia, the use of macrolides (31.3 %) for unapproved indications has also been reported.^29^ According to the WHO, the inappropriate use and overuse of antimicrobials are the main risk factors for the development of drug resistance.^9^ Thus, the WHO recommends various interventions to promote the rational use of antimicrobials, including the use of clinical practice guidelines, continuing medical education, the establishment of drug and therapeutic committees, supervision and auditing, among others.^10^

The main unapproved use of penicillins in this study was for the management of acute rhinopharyngitis, a condition that is generally secondary to viral infections.^30^^,^^31^ This finding is consistent with previous findings reported in Colombia, where antibiotics were used in 24.8 % of patients with viral infections of the upper respiratory tract, and the prescription of penicillins was widely predominant (73.3 %).^24^ The use of penicillins in purulent skin and soft tissue infections was also common. A study in Colombia revealed that 82.0 % of purulent infections are improperly managed.^15^ The Colombian clinical practice guidelines recommend the empirical use of penicillins for purulent methicillin-resistant *Staphylococcus Aureus* (MRSA) infections due to its high prevalence.^32^ However, no penicillin has a favorable effect on MRSA.^5^^,^^6^^,^^32^ Penicillins are also used for the management of urinary tract infections; however, their empirical use is not recommended because of increasing resistance to these medications in gram-negative bacilli such as *Escherichia coli*, which are the most common etiological agents of urinary tract infections.^33^^,^^34^ The literature describes multiple barriers that can lead to low adherence to the recommendations of clinical practice guidelines.^35^^,^^36^ For example, lack of knowledge of guidelines, lack of training, lack of time, lack of specialized personnel, and patients' sociocultural beliefs, among others.^35^^,^^36^

*Helicobacter pylori* infection was the most common approved indication in this study. This finding is consistent with that reported in a Colombian study on *Helicobacter pylori* eradication schemes, where amoxicillin was predominantly used (91.1 %).^16^ Clinical practice guidelines recommend treatment regimens that involve two or three antibiotics combined with a proton pump inhibitor and, in many cases, bismuth salts.^37^^,^^38^ The extensive use of amoxicillin in eradication schemes is because *Helicobacter pylori* continues to have good sensitivity to amoxicillin in Colombia (resistance 1.9 %‒9.5 %), in contrast to other antibiotics, such as nitroimidazoles or fluoroquinolones, where resistance is common (72.0 %‒88.0 % and 11.8 %‒27.3 %, respectively).^39^ However, eradication regimens containing metronidazole or levofloxacin were common. The problem previously identified in Colombia persists,^16^ which would go against the recommendations of the country's clinical practice guidelines.^38^ Finally, it is important to confirm the eradication of the microorganism once the treatment regimen is completed.^37^

Some variables that increased the probability of receiving penicillins for unapproved indications were identified. Younger patients from dispersed regions and prescriptions given by general medicine practitioners increased the risk of receiving penicillins for unapproved indications, which has already been observed in other real-world studies in Colombia.^29^ Variations in prescriptions between regions may be due to differences in the academic training of physicians, their prescription habits, the local epidemiology of infections, and the availability of medications.^29^ In Colombia, general practitioners reportedly have low levels of knowledge, attitudes and practices related to antibiotics, contributing to the inappropriate use of these drugs.^40^ Similarly, patients with a diagnosis of skin and soft tissue infections and infections of the lower respiratory tract were at greater risk of being improperly managed. This increased risk is due to inadequate antibiotic efficacy against MRSA in purulent skin and soft tissue infections^5^^,^^6^^,^^32^ and the use of antibiotics in infections that are usually of viral etiology, such as acute bronchitis and bronchiolitis.^5^^,^^6^^,^^31^ Patients who had recently used antibiotics were more likely to receive penicillins for unapproved indications. This result may reflect an uncertain diagnosis, antimicrobial resistance, poor adherence to clinical practice guidelines and even the demand for antibiotics by the patient.^11^

Some limitations should be considered in the interpretation of the results. Antibiotics purchased by patients with their own money are not included in the drug dispensing database of the pharmaceutical manager. The diagnosis for which penicillin was prescribed could not be identified for all patients because the database includes information only on the main diagnosis. The patients’ clinical history was not entered, and the patients were not contacted to document the diagnostic tests they received or to verify the diagnoses and their severity. It was also not possible to determine whether the antibiotic was used for the treatment of infections or for prophylaxis. Among the strengths of the study are the large sample size, the extensive distribution of patients throughout the national territory and the inclusion of patients from the two affiliation regimes of the country's health system.

## Conclusions

In this study, penicillins were prescribed mainly by general practitioners, and amoxicillin was the most common penicillin used in outpatients, followed by dicloxacillin, with oral presentations in tablets or capsules. In addition, approximately one-tenth of the patients received another antibiotic, particularly macrolides, and analgesics and anti-inflammatory agents were frequently used. The most common infections for which penicillins were prescribed were those of the upper respiratory tract and gastrointestinal tract, but one-third-of patients received prescriptions for indications that are not approved by regulatory agencies or clinical practice guidelines, especially patients with skin infections, purulent soft tissue infections or lower respiratory tract infections and those who recently used antibiotics.

Finally, these results highlight the need to improve pharmacovigilance and rational antibiotic prescribing systems. It is essential to review and disseminate updated evidence-based clinical guidelines adapted to the local context and the sensitivity and resistance results identified by healthcare institutions. It is important to implement continuing medical education programs and incorporate training in antimicrobial resistance and rational antibiotic use from the undergraduate level. Other strategies include prescription audits and feedback to physicians, as well as the implementation of digital tools in electronic medical records that alert physicians to unindicated prescriptions or recommended alternatives.

### Institutional review board statement

The protocol was approved by the Bioethics Committee of the Universidad Tecnológica de Pereira in the category of risk-free research. The ethical principles established by the Declaration of Helsinki were respected. Reference n° 14–130,223. The laws in Colombia (Resolution 8430 of 1993 of the Ministry of Health) exempt the obtaining of informed consent for risk-free research whose information is obtained from electronic records.

### Informed consent statement

No applicable, is a retrospective observational study.

### Data availability statement

(name of repository)

Protocolos.io.

## Authors’ contribution

LFVR participated in the drafting, data collection, data analysis, description of results, and discussion. BSAC participated in the drafting, description of results. LMLT participated in the drafting, description of results. MVJL participated in the drafting, description of results. MOR participated in the drafting, description of results. JEMA participated in the drafting, data collection, data analysis, description of results, discussion, critical revision of the article, and evaluation of the final version of the manuscript.

## Funding

This study did not receive funding.

## Conflicts of interest

The authors declare no conflicts of interest.

## Data Availability

Private link for reviewers: to be removed before publication https://www.protocols.io/private/F908CA8C7D1F11EF93050A58A9FEAC02.

## Appendix Supplementary materials

Image, application 1
