# Supplementary material for: Indications and prescriptions of penicillins in a population of Colombia: A cross-sectional study
Source: Braz J Infect Dis. 2025 Jul 16;29(5):104572. doi: 10.1016/j.bjid.2025.104572 (PMC12284274; doi:10.1016/j.bjid.2025.104572)
Supplement: Supplementary file 1 [file mmc1.docx]

**BJID-D-25-00039_Supplementary Material**

**Supplementary Table 1** Classification of the geographical regions of Colombia according to National Administrative Department of Statistics (DANE).

| **Regions** | **Departments** |
| --- | --- |
| Bogotá-Cundinamarca Region | Cundinamarca |
| Caribbean Region | Atlántico, Bolívar, Cesar, Córdoba, La Guajira, Magdalena, Sucre, San Andrés, Providencia and Santa Catalina. |
| Central Region | Antioquia, Caldas, Quindío, Risaralda, Caquetá, Huila, Tolima. |
| Pacific Region | Cauca, Chocó, Nariño, Valle del Cauca. |
| Eastern and Amazon-Orinoquía Region | Boyacá, Meta, Norte de Santander, Santander, Arauca, Casanare, Amazonas, Guaviare, Guainía, Vaupés, Vichada, Putumayo. |

**Supplementary Table 2** Approved indications for each penicillin.

| **Antibiotic** | **Indication** |
| --- | --- |
| Amoxicillin | Ear, nose and throat infections (otitis media, sinusitis, pharyngitis, tonsillitis), *Helicobacter pylori* eradication, lower respiratory tract infection (community-acquired pneumonia, bronchiectasis), actinomycosis, anthrax, asplenia (prophylaxis in patients at high risk), endocarditis (prophylaxis), Lyme disease (*Borrelia spp*. infection), periodontitis. |
| Amoxicillin/Clavulanate | Ear, nose and throat infections (otitis media, sinusitis, pharyngitis, tonsillitis, tonsillar abscess), lower respiratory tract infections (community-acquired pneumonia, acute exacerbation of bronchiectasis, acute exacerbation of chronic obstructive pulmonary disease), skin and subcutaneous tissue infections (impetigo, cellulitis/erysipelas, animal or human bite wounds, diabetic foot infection), community-acquired mild to moderate intra-abdominal infection without risk factors, and odontogenic infection |
| Ampicillin | Infections of the gastrointestinal tract (caused by *Shigella, Salmonella spp., Escherichia coli, Proteus mirabilis* and *enterococci*), infections of the genitourinary tract, and infections of the respiratory tract (caused by *Haemophilus influenzae, staphylococci* and *streptococci*). |
| Ampicillin/sulbactam | Infections of the skin and subcutaneous tissue, infections of the upper and lower respiratory tract, infections of the gastrointestinal tract. |
| Dicloxacillin | Infections caused by penicillinase-producing *staphylococci*. |
| Benzathine penicillin G | Congenital syphilis, syphilis (primary, secondary, early latent, late or of un-known duration), Sydenham chorea (prophylaxis), rheumatic fever (prophylaxis), acute glomerulone-phritis (prophylaxis), streptococcal pharyngitis/tonsillitis, endemic treponematoses (bejel, pinta, yaws). |
| Procaine penicillin G | Congenital syphilis, syphilis (primary, secondary or late), neurosyphilis (associ-ated with probenecid), diphtheria due to *Corynebacterium diphtheriae*, bacterial infection of the upper respiratory tract (*streptococcal tonsillitis*), *Streptococcus pyogenes* infection, group A streptococcal infection, erysipelas, necrotizing ulcerative gingivitis, spirochetosis, rat bite fever, scarlet fever, and endemic treponematoses (bejel, pinta, yaws). |
| Penicillin V | Bacterial endocarditis (prophylaxis in patients with congenital or acquired heart disease), Sydenham chorea (prophylaxis), rheumatic fever (prophylaxis), fusospirochetosis (mild to moderate), otitis media (mild to moderate), pneumococcal infection of the respiratory tract (mild to moderate), strep throat, gingivitis, odontogenic infection, uncomplicated rat bite fever, and upper respiratory infection (mild to moderate). |
